# Clinical Accuracy of the Lateral-Anterior Drawer Test for Diagnosing Posterior Cruciate Ligament Rupture

**DOI:** 10.1186/s40798-022-00500-4

**Published:** 2022-08-19

**Authors:** Gesine H. Seeber, Christoph Thalhamer, Omer C. Matthijs, Wolfgang Doskar, Phillip S. Sizer, Djordje Lazovic

**Affiliations:** 1 University Hospital for Orthopaedics and Trauma Surgery Pius-Hospital, Medical Campus University of Oldenburg, Georgstr. 12, 26121 Oldenburg, Germany; 2 Gelenkspezialisten, Specialist Center for Orthopedics, Trauma Surgery and Rehabilitation, Vienna, Austria; 3 Orthopaedic and Physical Therapy Outpatient Clinic Medzentrum23, Vienna, Austria; 4 BOMA, Physical Therapy Outpatient Clinic, Kapfenberg, Austria; 5 Trauma Center Klagenfurt Am Wörthersee, Klagenfurt, Austria; 6 Center for Rehabilitation Research, School of Health Professions, Texas Tech University Health Sciences Center, Lubbock, TX USA

## Abstract

**Background:**

Commonly used clinical posterior cruciate ligament (PCL) tests present with diagnostic weaknesses requiring alternative clinical tests. The Lateral-Anterior Drawer test (LAD-test) is a suggested alternative that previously demonstrated concurrent validity in situ*.* Further in vivo LAD-test clinical accuracy examination is required prior to any recommendation for clinical adoption. Thus, this case–control study aims to (1) investigate the LAD-test’s in vivo interrater and intra-rater reliability; (2) establish LAD-test concurrent validity against MRI as the reference standard; and (3) examine the correspondence between examiners’ professional working experience and LAD-test diagnostic accuracy.

**Methods:**

Three examiners with different professional experience levels, blindfolded during testing, and blinded from subjects’ identity, medical history, and reference test outcome performed all LAD-testing twice per subject. Reliability analyses included percent agreement, Fleiss’ kappa and Cohen’s kappa coefficients with 95% Confidence Intervals (CIs) and prevalence-adjusted bias-adjusted kappa (PABAK) calculations. Validation parameters included sensitivity, specificity, likelihood ratios (LR + ; LR-), and predictive values (PPV; NPV) each accompanied by 95%CIs; each tester’s percent agreement with the MRI; and their Youden Index.

**Results:**

The study sample was comprised of 31 subjects of which 14 had a history of unilateral full-thickness PCL-rupture. Their 14 contralateral knees and both knees of 17 healthy subjects served as controls. In vivo LAD-test performance did not produce any negative ramifications for the tested subjects. Interrater reliability was moderate (test-1: Fleiss’*κ* = 0.41; 95% CI 0.40;0.41; test-2:Fleiss’*κ* = 0.51; 95% CI 0.50;0.51). Pairwise examiner’s LAD-test outcome agreement ranged from 74 to 89%. Pairwise interrater reliability was fair-to-substantial (*κ* = 0.27 to *κ* = 0.65) with moderate-to-substantial PABAK (0.48–0.77). Intra-rater reliability was substantial-to-almost perfect (PABAK 0.65–0.97). Sensitivity and specificity ranged from 57 to 86% and 83 to 98%, respectively. The advanced and novice clinicians’ Youden Indexes were acceptable. The same examiners’ positive likelihood ratios revealed important and relative important effects, respectively. Positive predictive values were considerable for the advanced and novice clinicians, while negative predictive values were high for all examiners.

**Conclusion:**

Overall, the study results suggested LAD-test practicability. In vivo LAD-test performance did not produce any negative ramifications for the tested subjects. In subjects presenting with a chronic PCL-deficiency (i.e., > 3 months since initial injury), the LAD-test’s clinical accuracy was comparable-to-superior to other commonly used clinical PCL-tests. Future studies to establish the LAD-test’s usefulness in isolation as well as in combination with other clinical tests for acute PCL-rupture diagnostics are warranted.

**Trial registration number:**

DRKS00013268 (09. November 2017).

## Key Points

- This was the first study to establish the diagnostic accuracy for the novel LAD-test in vivo. Our findings suggest LAD-test feasibility and its usefulness for PCL-integrity detection in the clinical setting.
- The LAD-test provides manual feedback regarding ligament integrity associated with tibial translation and could be clustered with other PCL-tests to increase clinicians’ confidence in clinically detecting PCL-injuries.

## Background

Posterior cruciate ligament (PCL) injuries were long given limited consideration regarding diagnostics research [1]. This stems from a poor understanding of the PCL’s anatomic and biomechanical complexities and functional roles [1, 2]. Although the PCL´s importance is currently acknowledged [3–5], PCL-rupture prevalence is still underestimated; likely due to subtle, often unspecific signs and symptoms accompanying the acute injury [6, 7]. Thus, the estimated number of unidentified, chronic PCL-deficient knees is expected to be considerable [8].

Clinical tests are the mainstay of primary PCL-diagnostics [2, 6, 9, 10]. Clinicians commonly use the following clinical PCL-integrity tests: (1) posterior drawer test, (2) posterior sag sign, and/or (3) quadriceps active test [1, 2, 5–7, 10]. However, each present with specific diagnostic weaknesses [2, 11–13], meriting more precise clinical testing [1, 11, 14]. Further information demonstrating the clinical accuracy of currently available clinical PCL tests that supports our consideration can be witnessed in Kopkow et al.[11].

The Lateral-Anterior Drawer test (LAD-test) serves as an alternative approach, potentially resolving other PCL-tests’ limitations [15, 16]. The LAD-test is manually applied to a 90° flexed knee, positioning the PCL at near-perpendicular to the tibial plateau, where it controls lateral tibia-on-femur movements [15, 17–20]. The LAD-test force is applied medial-posterior–to–lateral-anterior to minimize intercondylar eminence constraint [15, 21]. The LAD-test’s surplus value is that it neither requires exact anatomical landmark palpation nor sufficient muscular relaxation, because it eludes any muscle’s functional plane. Moreover, the load is not applied in the instability’s direction, which could potentially reduce patient testing apprehension [15].

The LAD-test has demonstrated in situ construct and concurrent validity for detecting PCL-ruptures [15]. Whether this test reliably detects PCL-deficiency in vivo is unclear. This study aims to examine: (1) The LAD-test´s in vivo interrater and intra-rater reliability; (2) Concurrent validity between the LAD-test versus magnetic resonance imaging (MRI) as the reference; and (3) The correspondence between examiner professional working experience and LAD-test diagnostic accuracy. We crafted the following hypotheses: first, that the LAD-test will exhibit good intra-rater (PABAK > 0.61) and reasonable interrater reliability (PABAK > 0.41) in vivo (Hypothesis 1; H1); second, that the LAD-test will demonstrate acceptable concurrent validity (Youden Index ≥ 50%) relative to MRI (Hypothesis 2; H2); and third, that absolute agreement between LAD-test outcome and MRI outcome will not be significantly different amongst testers with different levels of professional working experience (*p* > 0.05; Hypothesis 3; H3).

## Methods

### Study Design

This case–control type clinical accuracy phase-II study [22] was conducted between November 2019 and July 2020 at a trauma center and two outpatient orthopedic clinics in central Europe. To address the study’s aims and hypotheses we incorporated an in vivo intra-rater and interrater reliability design and established LAD-test concurrent validity by testing for sensitivity, specificity, positive and negative predictive values (PPV and NPV, respectively), and positive and negative likelihood ratios (LR + and LR-, respectively) as well as LAD-test outcome percent agreement relative to MRI. Three examiners (all licensed physical therapists and certified orthopedic manual therapists) with different professional work experience records (7 [novice], 18 [advanced], and 44 [expert] years) performed all LAD-testing.

### Ethical Considerations

All study procedures followed the Declaration of Helsinki ethical principles. Ethical approval for study conduct was retrieved from the responsible ethics committees prior to initiation. Prior to subject recruitment, the study was registered in the German Clinical Trials Register and the study protocol was published [21]. All subjects gave their written informed consent for study participation.

### Sample Size Calculation

An a priori sample size calculation was performed based on Donner and Rotondi [23]. Current epidemiological data suggest a 4 to 40% PCL-rupture prevalence [13, 24–27]. Hence, a 25% PCL-rupture prevalence was estimated adequate in a diagnostic phase-II study sample. Based on this and a hypothesized 95% Confidence Interval (CI) lower limit of *κ* > 0.6, at least 55 knees were needed to determine a clinically meaningful interrater agreement. We sought a total sample of 30 subjects (60 knees); 15 subjects with a history of MRI-confirmed unilateral PCL-rupture (totaling 30 knees) plus 15 subjects with MRI-confirmed bilateral intact PCLs (totaling 30 knees).

### Inclusion and Exclusion Criteria

Eligible male and female PCL-subjects met the following inclusion criteria: (1) Age > 18 years; (2) acute (< 3 months since initial injury) or chronic (> 3 months since initial injury) MRI-confirmed unilateral complete isolated or combined PCL-rupture [28], and (3) first-time PCL-rupture. The following exclusion criteria precluded subject participation: (1) inability to sufficiently understand spoken and written German; (2) total knee arthroplasty; (3) previous ACL and/or PCL operations; (4) history of neurological conditions that may impair lower extremity function (e.g., spasticity); (5) any lower limb joint restrictions limiting the possibility to achieve the LAD-testing position; (6) inability to lie supine; (7) MRI contraindications; and (8) current pregnancy. Initially enrolled subjects who demonstrated difficulty in undergoing MRI (e.g., due to claustrophobia) precluded participation. Testing was discontinued if subjects experienced severe knee joint pain during LAD-testing.

### Pre-testing Procedures

**Fig. 1:**
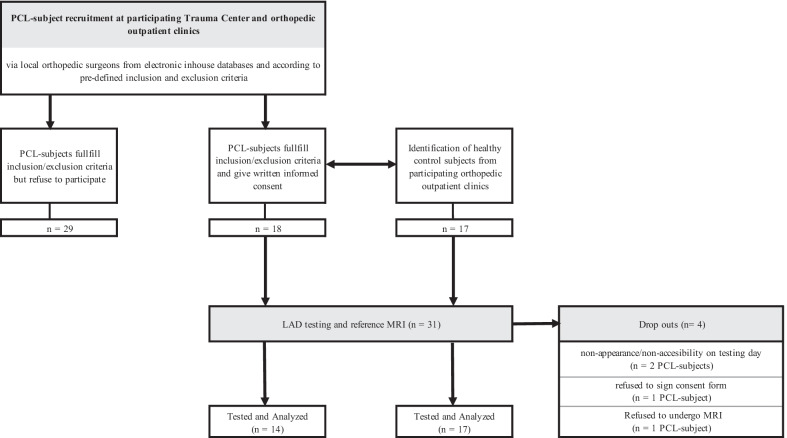
Participants’ flow through the study; PCL = Posterior Cruciate Ligament; LAD = Lateral-Anterior Drawer test (index test); MRI = Magnetic Resonance Imaging (reference test)

Healthy control-subjects were recruited once all PCL-subjects were identified (Fig. 1). Detailed study procedures have been previously described [21]. Investigators strove for consistency in subject scheduling, but limited flexibility was exercised to accommodate subject scheduling challenges. All subjects were randomly tested twice per examiner during data collection (test session-1 and test session-2). Examiners were blinded to participants´ history and MRI outcomes until data collection was completed. Examiners’ testing order was randomized using a computer-generated random number list. Examiners were blindfolded during testing.

### Testing Procedure

Before data collection, participants were instructed about all relevant study procedures. All subjects and examiners were gathered in adjacent holding rooms, so that examiners and subject groups had no contact throughout the entire data collection process. No communication was allowed within each group while in those rooms. For LAD-testing, participants randomly entered the examination room. Subjects’ age, sex, height, and weight information were entered on a consecutively numbered identification form before the subject self-positioned on the treatment table. Blindfolded examiners randomly entered the examination room one after the other and performed the LAD-test on the subject´s knees. Each examiner recorded their LAD-test outcomes independently on an individual case report form (CRF) after returning to their holding area.

### Reference Test

In response to the ethical challenges associated with using arthroscopy or functional radiographs in healthy subjects, a MRI reference standard was used by incorporating the following sequences: (1) sagittal T2-weighted; (2) sagittal Proton Density(PD) weighted, fat-saturated(fs); (3) coronal PD-weighted fs; and (4) axial PD-weighted fs. The blinded radiologist was experienced in knee MRI examination. Each MRI was performed in close proximity to clinical LAD-test data collection.

### Index Test

**Fig. 2:**
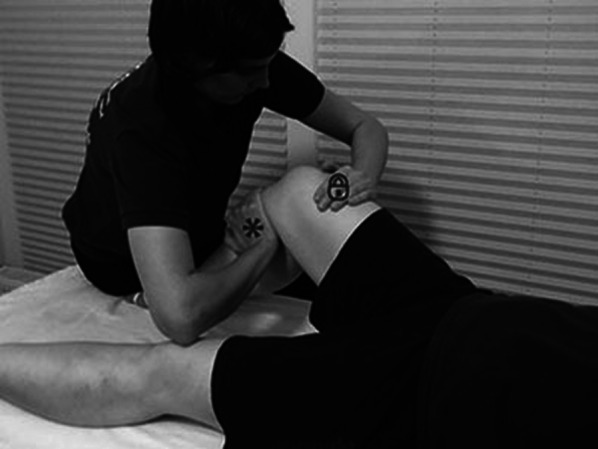
Performance of the Lateral-Anterior Drawer Test; * medial arm pushing proximal tibia in lateral-anterior direction; lateral hand stabilizing the femur in a medial-posterior direction. © Seeber GH et al. Cadaveric evaluation of the lateral-anterior drawer test for examining posterior cruciate ligament integrity. *Int J Sports Phys Ther*. 2017;12:569–580 (used with permission)

The supine LAD-testing position incorporated 45° hip flexion, 90° knee flexion, and a self-selected neutral tibial rotation [15–17]. The examiner fixed the participant’s lower limb by slightly sitting on the planted forefoot. The examiner placed one hand onto the femur´s lateral distal end without deforming the iliotibial band complex. The examiner fully pronated their other forearm and placed the heel of their hand onto the posterior-medial proximal tibia with the forearm oriented towards the anterolateral tibial tubercle (Fig. 2). The examiner moved the tibia back and forth through the available range of motion, in a medial-posterior–to–lateral-anterior direction, thereby noting the amount of lateral-anterior motion from the medial-posterior starting point. Following, the examiner repeated the LAD-test on the contralateral knee. After testing both knees the examiner rated each knee dichotomously as either ‘PCL-intact’ versus ‘PCL-deficient’, documenting the results on the aforementioned CRF. Each examiner was allowed to change back and forth between both knees repeating the LAD-test to raise their diagnostic certainty.

### Data Analyses

Data were analyzed using SPSS (Version 26; IBM Corp; Armonk, NY, USA). Descriptive statistics were obtained for overall sample characteristics. Central tendencies and dispersion data were established for the sample and each group regarding height, weight, BMI, age, and sex.

To address H1, Fleiss' kappa *κ* values with 95% CI were established to evaluate interrater reliability for the three examiners. Intra-rater and pairwise interrater reliability were calculated using Cohen’s kappa coefficients with 95% CI and percent agreement [29, 30]. A value of *κ* < 0.00, 0.00–0.20, 0.21–0.40, 0.41–0.60, 0.61–0.80, and 0.81–1.00 indicated poor, slight, fair, moderate, substantial, and almost perfect agreement, respectively [29]. As percent agreement does not take into account chance agreement [31] and Cohen´s kappa can be influenced by prevalence and between-examiner bias [32], using additional prevalence-adjusted bias-adjusted kappa (PABAK) has been recommended for more precise interpretation [33]. We calculated PABAK with data prevalence and bias indexes for both interrater and intra-rater reliability.

To address H2, percent agreement between LAD-test outcome versus MRI outcome was calculated. Moreover, the LAD-test’s sensitivity, specificity, LR + , LR-, PPV, and NPV with 95% CI were calculated using 2 × 2 tables [34]. Next, the Youden Index was calculated for each examiner. This measure takes both sensitivity and specificity into account, which are the two recommended statistical parameters to inform about the level of agreement for dichotomously rated outcomes [35]. It ranges from zero to 100%; where the closer the measure is to 100% the greater is the test’s discriminative power. The agreed minimum acceptable value is 50% [36]. The following equation was used to calculate the Youden Index: (Sensitivity% + Specificity%) − 100 [36].

To address H3, a generalized linear mixed model using a logit link function and a binomial probability distribution (logistic regression with random effect) was incorporated for examiner pairwise comparison. The dependent variable was the agreement of the index test (i.e., LAD-test) and the reference test (i.e., MRI). Fixed influencing factor was examiner (expert, advanced, and novice). The dependent data structure (two knees per subject, two evaluations of each examiner per knee, and three examiners) were model by including a random intercept for knee within subject. An additional random intercept for subject could not be estimated, so the final model was reduced including a random intercept for knee within subject only. The empirical robust covariance estimator was applied and the significance level was set to 0.05.

Two of the control knees’ reference MRI revealed incidental findings of minor PCL-deficiency. Therefore, agreement between examiners’ LAD-test outcome versus MRI and LAD-test diagnostic properties were additionally analyzed using an as-treated analysis, where subjects’ initial allocation was no longer respected. Rather, those two control knees became classified as additional PCL-deficient knees. Subsequent calculations followed the same procedures as described above.

## Results

### Sample

Eighteen PCL- and 17 healthy control-subjects participated in the study. Four PCL-subjects disenrolled due to non-appearance (*n* = 2), reluctance to consent (*n* = 1), and refusal for MRI (*n* = 1). Thus, the final sample included 14 PCL-deficient and 17 control-subjects. The 19 male and 12 female subjects exhibited a mean age of 36 ± 12 years. Subjects’ mean height and weight were 175 ± 1 cm and 78.6 ± 12 kg, respectively, with a mean BMI of 25.5. Table 1 reports group-specific demographics.

**Table 1:** Characteristics of PCL-subjects and control-subjects

		PCL-subjects (*n* = 14)	Control-subjects (*n* = 17)
Sex			
	Male (*n*; %)	11; 78.6	8; 47.1
	Female (*n*; %)	3; 21.4	9; 52.9
Age (years; mean ± SD)		35 ± 16	37 ± 9
Body height (cm; mean ± SD)		178 ± 1	172 ± 1
Body weight (kg; mean ± SD)		86 ± 11	72 ± 10
BMI (mean ± SD)		27 ± 4	24 ± 2
Unilateral PCL-rupture			
	Right (*n*; %)	8; 57.1	
	Left (*n*; %)	6; 42.9	

SD = Standard deviation, PCL = Posterior cruciate ligament, BMI = Body Mass Index

All PCL-subjects were initially diagnosed with a full-thickness PCL-rupture, as per medical record. All PCL-subjects presented at enrollment with a chronic PCL-lesion. The shortest and longest interval from initial rupture diagnosis to study enrollment was three months and 20 years, respectively. The mean period from initial PCL-rupture diagnosis to this study’s reference MRI was 2.4 ± 5 years. No subject reported any negative ramifications (e.g., knee pain, major discomfort, or knee joint swelling) during and/or after LAD-testing.

This study’s reference MRI showed residual PCL-deficits in all PCL-ruptured knees (*n* = 14). However, some exhibited variable healing levels (e.g., partial scarring). Adapted from the American Academy of Orthopedic Surgeons ligamentous injuries classification scheme, MRI-findings revealed that 5 (36%) PCL-deficient knees still demonstrated a Grade-3 PCL-injury, 5 (36%) showed a Grade-2 PCL-injury, and 4 (28%) showed a Grade-1 PCL-injury. Control knees’ MRI findings exhibited healthy PCL presentations with two exceptions as previously reported. None of the knees presented with MRI-confirmed posterior lateral corner injuries. Evidence of scarring was observed in five medial collateral ligaments (MCL) ipsilateral and two MCL contralateral to the subjects’ PCL-involved knees. Two control subjects failed to report an ACL reconstruction history at the time of enrollment, which surfaced in the study MRI. Yet, these two control subjects were not dismissed for the following reasons: (1) A lack of any other tissue compromise on the study MRI; and (2) a negative PCL-deficiency finding would speak to the LAD-test’s direction-specific nature. No enrolled PCL-subject was dismissed in response to exclusion criteria.

### Hypothesis 1–Testing

#### Interrater Reliability

Interrater reliability between all examiners was moderate at both the first (Fleiss´*κ* = 0.41; 95% CI 0.40;0.41) and second LAD-test sessions (Fleiss’*κ* = 0.51; 95% CI 0.50;0.51). Pairwise interrater reliability for each test session is shown in Table 2. A 74% and 77% agreement between the expert and novice clinician accompanied by moderate PABAK findings (0.48 and 0.55) were witnessed during test sessions-1 and -2, respectively. A 76% and 85% agreement were observed between the expert and advanced clinician during test sessions-1 and -2. These were accompanied by moderate-to-substantial PABAK findings (0.52 and 0.71) for the two test sessions, respectively. An 89% and 85% agreement were observed between the advanced and novice clinician during test sessions-1 and -2, respectively. Such was accompanied by substantial PABAK findings (0.77 and 0.71).

**Table 2:** Pairwise LAD-test interrater reliability during test sessions-1 and -2

	Test session-1						Test session-2					
	Agreement (%)	Cohen's kappa	PABAK	Bias index	Prevalence index	PABAK Indicates	Agreement (%)	Cohen's kappa	PABAK	Bias index	Prevalence index	PABAK Indicates
Expert versus. advanced clinician	76	0.33	0.52	0.05	− 0.53	moderate agreement	85	0.57	0.71	− 0.02	− 0.56	substantial agreement
Expert versus novice clinician	74	0.27	0.48	0.06	− 0.55	moderate agreement	77	0.36	0.55	− 0.03	− 0.55	moderate agreement
Advanced versus novice clinician	89	0.65	0.77	− 0.02	− 0.60	substantial agreement	85	0.60	0.71	0.02	− 0.53	substantial agreement

PABAK = Prevalence-adjusted, bias-adjusted kappa

#### Intra-Rater Reliability

The LAD-test outcomes agreement between test sessions-1 and -2 for the expert, advanced, and novice clinicians were 82%, 98% and 89%, respectively. Intra-rater reliability for the same clinicians was moderate (*κ* = 0.51), almost perfect (*κ* = 0.95), and substantial (*κ* = 0.67), respectively. Finally, PABAK suggested substantial (0.65), almost perfect (0.97), and substantial (0.77) intra-rater reliabilities for the expert, advanced, and novice clinicians, respectively (Table 3).

**Table 3:** Intra-rater reliability of the three different examiners

	Agreement (%)	Cohen's kappa	PABAK	Bias index	Prevalence index	PABAK Indicates
Expert clinician	82	0.51	0.65	0.05	− 0.53	Substantial agreement
Advanced clinician	98	0.95	0.97	− 0.02	− 0.56	Almost perfect agreement
Novice clinician	89	0.67	0.77	− 0.05	− 0.56	Substantial agreement

PABAK = Prevalence-adjusted, bias-adjusted kappa

### Hypothesis 2–Testing

#### LAD-Test Concurrent Validity

Table 4 provides information regarding LAD-test concurrent validity as compared to MRI for test sessions-1 and -2 and all examiners. Per-protocol analyses revealed good (> 75%) agreement between LAD-test outcomes and MRI findings for each clinician during both test sessions. The same analysis revealed the following LAD-test diagnostic accuracy results: Sensitivity between 57 and 86%; Specificity between 83 and 98%; LR + between 3.43 and 41.14; LR- between 0.15 and 0.51; PPV between 50 and 92%; and NPV between 87 and 96%. While the novice and advanced clinicians’ Youden Indexes reached and exceeded the accepted 50% value, the expert clinician’s Youden Index remained below the targeted threshold (Table 4). All information regarding agreement between each examiner’s LAD-test outcome and the MRI, and LAD-test diagnostic accuracy values with corresponding 95%CI established during the as-treated analysis are provided in Table 4.

**Table 4:** LAD-test diagnostic properties as established by each examiner

	LAD-test diagnostic accuracy (per-protocol results)						LAD-test diagnostic accuracy (as-treated results)					
	Expert clinician		Advanced clinician		Novice clinician		Expert clinician		Advanced clinician		Novice clinician	
	**Test session-1**						**Test session-1**					
		95% CI		95% CI		95% CI		95% CI		95% CI		95% CI
Sensitivity	57%	29%; 82%	86%	57%; 98%	64%	35%; 87%	56%	30%; 80%	75%	48%; 93%	56%	30%; 80%
Specificity	83%	70%; 93%	98%	89%; 100%	94%	83%; 99%	85%	71%; 94%	98%	88%; 100%	93%	82%; 99%
PPV	50%	25%; 75%	92%	64%; 100%	75%	43%; 95%	56%	30%; 80%	92%	64%; 100%	75%	43%; 95%
NPV	87%	74%; 95%	96%	86%; 100%	90%	78%; 97%	85%	71%; 94%	92%	80%; 98%	86%	73%; 94%
LR +	3.43	1.57; 7.47	41.14	5.85; 289.53	10.29	3.21; 32.91	3.70	1.65; 8.29	34.5	4.86; 244.70	8.63	2.66; 27.97
LR-	0.51	0.28; 0.95	0.15	0.04; 0.53	0.38	0.19; 0.77	0.52	0.29; 0.91	0.26	0.11; 0.60	0.47	0.27; 0.82
	*Youden Index*						*Youden Index*					
	40%		84%		58%		41%		73%		49%	
	*Agreement with MRI findings*						*Agreement with MRI findings*					
	77%		95%		87%		77%		92%		84%	
	**Test Session-2**						**Test Session-2**					
Sensitivity	57%	22%; 82%	86%	57%; 98%	79%	49%; 95%	56%	30%; 80%	75%	48%; 93%	69%	41%; 89%
Specificity	90%	77%; 97%	96%	86%; 99%	92%	80%; 98%	91%	79%; 98%	96%	85%; 99%	91%	79%; 98%
PPV	62%	32%; 86%	86%	57%; 98%	73%	45%; 92%	69%	39%; 91%	86%	57%; 98%	73%	45%; 92%
NPV	88%	75%; 95%	96%	86%; 99%	94%	82%; 99%	86%	73%; 94%	92%	80%; 98%	89%	77%; 96%
LR +	5.49	2.13; 14.12	20.57	5.21; 81.24	9.43	3.55; 25.06	6.47	2.31; 18.14	17.25	4.32; 68.89	7.91	2.93; 21.34
LR-	0.48	0.26; 0.88	0.15	0.04; 0.54	0.23	0.09; 0.64	0.48	0.27; 0.84	0.26	0.11; 0.61	0.34	0.17; 0.71
	*Youden Index*						*Youden Index*					
	47%		82%		71%		47%		71%		60%	
	*Agreement with MRI findings*						*Agreement with MRI findings*					
	82%		94%		89%		82%		90%		85%	

LAD = Lateral-Anterior Drawer test; PPV = positive predictive value; NPV = negative predictive value; LR +  = positive likelihood ratio; LR- = negative likelihood ratio; MRI = Magnetic resonance imaging

95% CI = 95% Confidence Interval (lower; upper limit)

### Hypothesis 3–Testing

Per-protocol analysis revealed a significant overall effect for examiner (F[2, 369] = 4.508; *p* = 0.012). There was a statistically significant difference between the expert versus advanced clinicians’ absolute agreement between LAD-test outcome and MRI outcome (*p* = 0.004). The same analysis revealed no significant differences between the advanced versus novice clinicians’ and the novice versus expert clinicians’ results (Table 5). Additional information regarding the pairwise comparison results of the agreement between the index test versus reference test per examiner established during the as-treated analysis can be found in Table 5.

**Table 5:** Pairwise comparison of the agreement of the index test and reference test per examiner

Per-protocol Comparison^§^	Odds Ratio (95% CI)	Wald *p*-value	As-treated Comparison	Odds Ratio (95% CI)	Wald *p*-value
Advanced versus expert clinician^§^	5.259 (1.719–16.071)	0.004	Advanced versus expert clinician	3.313 (1.210–9.061)	0.020
Advanced versus novice clinician	2.581 (0.794–8.390)	0.115	Advanced versus novice clinician	2.164 (0.849–5.512)	0.106
Novice versus expert clinician	2.036 (0.848–4.889)	0.111	Novice versus expert clinician	1.531 (0.637–3.684)	0.340
Overall examiner effect: F(2, 369) = 4.508; *p* = 0.012			Overall examiner effect: F(2, 369) = 2.779; *p* = 0.063		

^§^Example: The odds for absolute agreement between the LAD-Test outcome vs. MRI outcome was 5.259 times higher for the advanced clinician compared to the expert clinician

95% CI = 95% Confidence Interval (lower; upper limit)

## Discussion

The current study was the first to examine the LAD-test diagnostic accuracy in vivo. Our results indicate a moderate overall interrater reliability. Prevalence-adjusted, bias-adjusted pairwise interrater reliability showed moderate agreement between the expert and novice clinicians and a moderate-to-substantial interrater reliability for the advanced clinician with both the expert and novice. LAD-test percent agreement was > 80% for all examiners, and their intra-rater reliability was substantial-to-almost-perfect. In addition, overall diagnostic accuracy of the LAD-test presents comparable-to-superior to other clinical PCL-tests.

A valid test helps to accurately confirm a disorder’s presence or absence [34]. The LAD-test’s sensitivity describes its ability to detect a PCL-injury when it is indeed present, while the test’s specificity describes its ability to obtain a negative test outcome when the PCL is truly intact. A diagnostic test’s feasibility relates to its clinical utility for providing an adequate number of correct responses, which is represented by its PPV and NPV [34, 37]. Here, the PPV estimates the likelihood that an individual presenting with a positive LAD-test actually has a PCL-injury, while the NPV estimates the probability that a person who tested negative actually does not have a PCL-injury [34]. Our speculation that the LAD-test would show acceptable concurrent validity relative to MRI found conflicting results. On the one hand, the LAD-test presented with reasonable specificity among all examiners, while test sensitivity was consistently lower and varied considerably among examiners. Considering the LAD-test’s acceptable specificity it can be concluded that clinicians can be fairly confident that a positive LAD-test represents an actual present PCL-lesion. However, one cannot draw a generalizable conclusion with respect to the diverse sensitivity data observed in this study.

The NPV was acceptable for all examiners (> 85%) suggesting that a high proportion of individuals with an intact PCL were correctly tested negative [37]. For the LAD-test’s PPV a similar picture was observed; PPV were relatively high for the novice and advanced clinicians, where between 73 and 92% of individuals who tested positive indeed had a radiologically confirmed PCL-lesion, implying that false positive LAD-test outcomes were minimal [37]. However, the expert clinician’s LAD-test PPV results were not equally impelling.

Although knowledge about any clinical test’s sensitivity, specificity, PPV, and NPV is important for test selection in the clinical setting, the LR + and LR- may provide the greatest value for clinicians [34]. Those can help raise or lower a diagnosis’ pre-test–to–post-test probability. Thus, they can help clinicians becoming more confident about their diagnostic hypothesis by informing them about how much more likely a disorder is present following test performance and outcome interpretation [34]. A high LR + indicates that a disorder is likely to be present with a positive test, while a very low LR- is warranted to indicate that there is only a very small probability left for the disorder to be present with a negative test outcome [34]. A LR +  > 10 and LR- < 0.1 are considered important effects, while values of LR +  > 5 and LR- < 0.2 can still be considered relatively important effects with regard to clinical knee ligament integrity evaluation tests [34]. The current study’s overall LR + and LR- data suggest that the LAD-test is helpful to rule in a PCL-injury with a positive test outcome, while in case of a negative test outcome the diagnosis cannot be ruled out with very high certainty.

Kopkow et al. [11] published a systematic review about PCL physical examination tests. From the commonly used clinical PCL-tests, the quadriceps active test appeared to be most specific, while the posterior sag sign seemed to be most sensitive [11]. Yet, nine out of the eleven studies informing Kopkow et al. [11] presented with high risk of bias, thus hampering conclusive PCL-test diagnostic accuracy interpretation.

Sensitivity values established by two out of three examiners in the current study ranged from comparable-to-superior compared to previously reported posterior sag sign sensitivity [38, 39]. The LAD-test’s specificity established by all current examiners appears broadly comparable to that of the posterior drawer test and posterior sag sign found by other authors [38, 39]. However, one must consider those authors’ investigations presented with modest risk for bias [11]. Based on results for all current study examiners, the LAD-test could be deemed more valuable than the quadriceps active test for correctly identifying individuals who truly have a PCL-injury [38]. While this study suggests similar LAD-test LR + and LR- values for the novice clinician compared to those previously reported for the quadriceps active test, the advanced clinician’s LAD-test likelihood ratios were superior [38]. Furthermore, with a negative test result the LAD-test overall seems to shift post-test probability more meaningful than the posterior sag sign [38].

It is important to note that this study’s results can only be applied to chronic PCL-deficient knees as any determination of the LAD-test’s diagnostic accuracy in acute PCL-deficient knees was not possible within this study´s sample. However, acute PCL-injury may be accompanied by apprehension or increased post-traumatic muscle guarding [5], thus hampering accurate posterior drawer test, posterior sag sign or quadriceps active test performance and/or interpretation [15, 39]. In contrast, no appreciable muscle relaxation is mandatory during LAD-test performance as its testing direction is outside any muscle’s functional plane and, in addition, the manual load is not applied in the knee’s direction of instability [15]. Moreover, the examiner’s manual tibial contact during LAD-test performance can provide the clinician with valuable tactile tissue integrity information associated with the tibial translation [15]. This may lead to superior LAD-test diagnostic accuracy in acute PCL-deficient knees. A prospective phase-III clinical accuracy study is warranted to further examine the LAD-test in acute PCL-deficient knees. Future studies investigating the LAD-test versus the commonly used clinical PCL-tests and/or different PCL-test clusters in the same sample of acute and/or chronic PCL-deficient knees, as well as in patients presenting with full-thickness tears versus partial PCL-rupture, are warranted. Moreover, future studies should examine the LAD-test’s performance—in isolation and/or combined with other clinical PCL-tests—in multiple ligament injured knees versus isolated PCL-tears. Such investigations would facilitate the comparison of each test’s diagnostic accuracy and the diagnostic accuracy of test clusters more completely and thus find the most useful clinical PCL-test or the best test combinations for specific patient groups.

In our H3 we hypothesized that different levels of professional working experience (in years) would not lead to LAD-test versus MRI agreement differences amongst testers. However, this hypothesis was not fully met. Although only the differences in absolute agreement between the expert versus the advanced clinician reached significance, both the advanced and novice clinicians performed superior compared to the expert clinician in all measures. Several explanations for these findings are conceivable: First, using years of professional working experience as licensed clinician to categorize the ‘expert’ versus ‘advanced’ versus ‘novice’ clinician may have not been the best choice. Because of the injury’s small incidence, most clinicians do not have the opportunity to frequently witness a PCL-deficient knee throughout their career [15]. Consequently, even clinicians who have a high number of professional working years may lack knowledge and hands-on practice regarding the feel of a clinical PCL-test with a truly injured ligament. In hindsight, we did not account for the depth of experience with PCL-deficient knees. The study may have been better served by categorizing the clinicians based on exposure to the actual numbers of PCL-deficient knees seen during their career. However, in retrospect that was impossible to quantify where it would have been speculative at best. Future studies should use more appropriate criteria to obtain clear discriminatory power between different experience levels.

A second possibility for LAD-test validity and reliability differences between different examiners may be the presence of cognitive biases and/or personality trait differences among testers that may affect a clinician’s decision-making processes [40]. For example, both the advanced and novice clinicians may have been less confident about their diagnostic skills. This, in addition to a lower tolerance for uncertainty or diagnostic ambiguity, may have led to a more extensive LAD-testing compared to the expert clinician [40]. Although, LAD-test performance was discussed amongst the group and practiced for standardization purposes prior to actual data collection, LAD-testing was not limited to a specific number of test repetitions per knee. Rather, in accordance with common LAD-test practices in the clinical setting, each tester was free to repeat the LAD-test more than once at each subject’s knees during their given testing session until they were confident about their findings. Experts on the contrary are often very confident about their diagnostic skills and findings, what may lead to less diagnostic accuracy [40]. In case of a clinical testing maneuver such as the LAD-test, overconfidence and premature closure bias may lead to a more rapid testing and subsequent decision-making process, where the clinician may misinterpret the outcomes out of haste when only subtle aberrant joint movement changes may be present. Unfortunately, no data have been collected on how often each tester actually performed the LAD-test on a respective subject’s knees during each test session and/or how confident each clinician felt during and after LAD-testing. Thus, no valid conclusion about the influence of individual cognitive bias and/or personality traits can be made on the current study’s results and should be evaluated in future investigations.

Finally, as the examiners were blindfolded throughout testing, they could only rely on sensory-motor perceptual judgement during their LAD-test performance. This is somewhat different from routine clinical testing where visual information of abnormal tibial movement can support the clinician’s diagnostic hypothesis of PCL-insufficiency. Moreover, physical examination and clinical decision-making is normally not based on only one single test but rather on a comprehensive patient history in combination with accumulated sensory-motor and visual perceptional information retrieved from different clinical testing maneuvers [12, 38]. Using only the LAD-test for decision-making may have been more or less challenging for each examiner.

## Limitations

This study followed a case–control design that is somewhat artificial as the study population is preselected, possibly facilitating spectrum bias. Moreover, all examining clinicians were aware of the study’s aim (i.e., attempting to evaluate their PCL ligament integrity examination accuracy using the LAD-test). Thus, examiners may have maintained a higher suspicion for PCL-injuries within the study sample. However, the central question of a diagnostic accuracy phase-II study is whether a clinical test in question (here the LAD-test) can sufficiently detect a disease’s presence in actual patients versus its absence in healthy individuals [22, 38]. In a previous diagnostic phase-I study Seeber et al. [15] established the LAD-test’s construct and concurrent validity in an experimental context. Only now, following the completion of the current diagnostic phase-II study it seems meaningful, based on current results, to further investigate the LAD-test in the clinical setting. Future experimentation should include less artificial samples and follow a prospective design. However, knowing about the inherent weaknesses of a case–control design, the team exercised measures in an attempt to best counteract possible biases. First, all examiners were completely blindfolded from before they entered until after they left the examination room. Second, examiner and participant appearances to the examination room were fully randomized. Third, no conversation was allowed between examiners and participants at any time during data collection, nor were examiners allowed to communicate with each other about their findings. Fourth, LAD-test results were not disclosed to the examiners until data collection was completed. Fifth, the involved clinicians were blinded to any radiological findings prior to data collection. Sixth and final, any participant’s LAD-test outcomes were not disclosed to the radiologist.

Another challenge in case–control studies is the influence of possible confounding factors [34]. With regard to knee ligament rupture these could include age, sex, height, and weight. Matching is one way to minimize the influence of potential confounders [34]. Therefore, in the present study we aimed to match subjects according to the aforementioned characteristics. However, this was not completely successful due to the premature disenrollment of four PCL-subjects as outlined above. Moreover, sporting history elements that could have contributed to ligament ruptures were not collected at the subjects’ entry into the study. Future studies that further examine the impact of the LAD-test should give this consideration.

Another limitation centers on the use of MRI as the reference test. While MRI has been shown to accurately detect acute PCL-ruptures [7, 41–47] a 100% accurate diagnosis of chronic PCL-injuries seems impossible [38, 46, 48]. However, all current PCL-deficient knees had to be classified as chronic as previously reported. Kneeling stress radiographs would be the ideal reference test for diagnosing chronic PCL-injuries [49–51]. However, such imaging technique was not approved by the responsible ethics commission. Furthermore, although all enrolled PCL-subjects had initially been diagnosed with a unilateral full-thickness PCL-tear, the reference MRI revealed some form of healing in 9 out of 14 PCL-deficient knees. While MRI can well distinguish between a full-thickness versus a partial-thickness tear versus an intact ligament, it is not as useful to rate the ligament’s quality or functionality [48]. Being able to rate a ligament’s functional status seems more important than knowing about how it appears on the MRI because a partial tear may either end up with a functional (i.e., still sufficiently stabilizing the knee joint) or non-functional PCL (i.e., not sufficiently stabilizing the knee anymore). In addition, any PCL-tear may heal completely, appearing intact on MRI, but still may present with insufficient functionality due to healing in incorrect length [48], or inadequate sensorimotor control recovery [52]. Thus, in case of a partially torn/healed PCL witnessed on MRI versus a positive or negative clinical PCL-test, one must ask which measure best represents the ligament’s functionality and is most important in the clinical setting—the MRI or the clinical test. Thus, functional imaging such as kneeling stress radiographs or diagnostic arthroscopy would have served as more useful reference tests. However, as the current study’s sample involved generally healthy participants, the use of such reference tests to objectively investigate PCL-integrity and ligament functionality at the same time was impossible as outlined before. Further prospective studies using more relevant reference tests are strongly warranted.

A third limitation may be the use of the Youden Index as the statistical parameter informing about the index test’s concurrent validity in relation to a reference test as it may not represent the complete picture of a test’s diagnostic accuracy. However, to our knowledge the Youden Index is the only measure of diagnostic accuracy with an agreed general minimum acceptable value [36]. Thus, in order to create a testable hypothesis regarding the LAD-test’s concurrent validity we report this measure. Yet, a disadvantage of the Youden Index is its lack of sensibility for differences in a test’s sensitivity and specificity [53]. Moreover, other measures of diagnostic accuracy such as positive and negative predictive values as well as likelihood ratios may be more informative regarding a clinical test’s utility and should thus be additionally taken into account.

## Conclusion

Overall, the study results suggested LAD-test practicability. In vivo LAD-test performance did not produce any negative ramifications for the tested subjects. The LAD-test showed a moderate agreement between multiple testers. Pairwise interrater reliability indicated moderate-to-substantial agreement between differently experienced clinicians. Intra-rater reliability was substantial-to-almost-perfect. In subjects presenting with a chronic PCL-deficiency (i.e., > 3 months since initial injury), the LAD-test’s clinical accuracy seemed comparable-to-superior to other clinical PCL-tests. Future studies should establish the LAD-test’s usefulness in isolation as well as in combination with other clinical tests for acute PCL-rupture diagnostics.

## Data Availability

All data relevant to the study are included in the article.

## Abbreviations

BMI: Body Mass Index
CRF: Case report form
LAD: Lateral-Anterior Drawer
LR-: Negative likelihood ratio
LR +: Positive likelihood ratio
MCL: Medial collateral ligament
MRI: Magnetic resonance imaging
NPV: Negative predictive value
PABAK: Prevalence-adjusted bias-adjusted kappa
PCL: Posterior cruciate ligament
PPV: Positive predictive value
